# Behavioural determinants shaping infection prevention and control behaviour among healthcare workers in Dutch general practices: a qualitative study reflecting on pre-, during and post-COVID-19 pandemic

**DOI:** 10.1186/s12875-024-02304-9

**Published:** 2024-02-28

**Authors:** Famke Houben, Casper DJ den Heijer, Mitch van Hensbergen, Nicole HTM Dukers-Muijrers, Eefje GPM de Bont, Christian JPA Hoebe

**Affiliations:** 1 Department of Social Medicine, Care and Public Health Research Institute (CAPHRI), Faculty of Health, Medicine and Life Sciences, Maastricht University, P.O. Box 616, Maastricht, 6200 MD The Netherlands; 2 Department of Sexual Health, Infectious Diseases and Environmental Health, Living Lab Public Health Mosa, South Limburg Public Health Service, P.O. Box 33, Heerlen, 6400 AA The Netherlands; 3 Department of Medical Microbiology, Infectious Diseases and Infection Prevention, Care and Public Health Research Institute (CAPHRI), Faculty of Health, Medicine and Life Sciences, Maastricht University Medical Centre (MUMC+), P.O. Box 5800, Maastricht, 6202 AZ The Netherlands; 4 Department of Health Promotion, Care and Public Health Research Institute (CAPHRI), Faculty of Health, Medicine and Life Sciences, Maastricht University, P.O. Box 616, Maastricht, 6200 MD The Netherlands; 5 Department of Family Medicine, Care and Public Health Research Institute (CAPHRI), Faculty of Health, Medicine and Life Sciences, Maastricht University, P.O. Box 616, Maastricht, 6200 MD The Netherlands

**Keywords:** SARS-CoV-2, Infection control, Primary health care, General practice, Family medicine, Behavioural determinants, Qualitative research

## Abstract

**Background:**

Since the Coronavirus Disease 2019 (COVID-19) pandemic, awareness of infection prevention and control (IPC) has increased in primary care settings. This study aimed to examine behavioural determinants shaping IPC behaviour pre-, during, and post-pandemic among healthcare workers (HCWs) in general practices, to inform optimised IPC in primary care.

**Methods:**

For this qualitative study, semi-structured in-depth interviews were conducted during two study periods: (1) pre-COVID-19 pandemic: July 2019-February 2020, with 14 general practitioners (GPs) and medical assistants, and (2) during the COVID-19 pandemic: July 2022-February 2023, with 22 GPs and medical assistants. The design was informed by behaviour change theories. Data were analysed using thematic analysis.

**Results:**

Main themes were: (1) risk perception and IPC awareness, (2) attitudes towards IPC and professional responsibility, (3) decision-making process and risk considerations for IPC adherence, (4) social norm and social influence in GP practice team, and (5) environmental context and resource availability in GP practice. During the pandemic, risk perception and awareness of the importance of IPC increased compared to the pre-pandemic period. A consistent belief emerged that IPC is part of professional responsibility, while needing to be balanced with other aspects of patient care. Decision-making is dependent on the individual GP and mainly influenced by risk assessments and sustainability considerations. The social context in the practice team can reinforce IPC behaviours. GP practice building and layout, and limited IPC resource and material availability were reported as main barriers.

**Conclusions:**

The theory-informed insights of this study can be used for targeted interventions to optimise IPC behaviour in general practices. Adopting multifaceted strategies to target the various determinants is recommended to sustain IPC, by implementing continuous education using tailored communication, integrating IPC in work routines and organisational workflows, refining existing IPC protocols by incorporating decision-making tools for HCWs, fostering a culture of IPC through knowledge-sharing and teamwork, and addressing GP practice physical environment and IPC resource barriers.

**Supplementary Information:**

The online version contains supplementary material available at 10.1186/s12875-024-02304-9.

## Background

The COVID-19 pandemic has put a strain on healthcare systems worldwide [1]. Still, it has also underscored the critical role of infection prevention and control (IPC) practices in safeguarding both patients and healthcare workers (HCWs) [2]. Primary care settings, including general practices, have been forced to reevaluate and adapt the organisation and implementation of IPC to meet the high demands of the pandemic [3–5]. Before the pandemic, IPC practices primarily encompassed fundamental hygiene measures such as hand hygiene, personal hygiene, sterile procedures, sterilisation of medical instruments, and disinfection of environmental surfaces [6]. However, with the onset of the pandemic, IPC measures intensified to mitigate transmission risks. These intensified IPC measures involved the increased use of personal protective equipment (PPE) such as face masks and gloves, the implementation of stringent triage protocols, the implementation of patient screening procedures, adjustments to patient flow management, modifications in practice layouts, and increased use of digital care [7–13]. HCWs including general practitioners (GPs) played a key role in driving these adaptations, and successful implementation of IPC relied on active involvement and behavioural changes to adopt and sustain IPC behaviours.

While the adoption of IPC practices has been paramount, there is scarce research on behavioural determinants that influence IPC behaviour among HCWs in general practices. Previous studies primarily focused on mapping organisational and procedural aspects of IPC, with a predominant focus on logistical factors influencing IPC during the pandemic. Evidence has shown enhanced adherence to IPC among HCWs during the COVID-19 pandemic compared with the pre-pandemic period [14–16]. It is expected that the felt urgency reflecting the risk and fear of infection and outbreaks heightens awareness of the importance of complying with IPC guidelines and drives positive changes in IPC behaviours of HCWs [17]. Before the pandemic, compliance of HCWs with IPC in Dutch general practices had substantial room for improvement [18], particularly regarding hand hygiene practices [19].

Acknowledging that IPC implementation relies on the behaviour of individuals, it is essential to understand the behavioural determinants shaping IPC practices from the experiences and perspectives of HCWs in general practices. For instance, behavioural determinants include HCWs’ attitudes and beliefs about the importance and effectiveness of IPC measures [17, 20]. The application of theory-based approaches, grounded in behaviour change theories, can facilitate identifying and understanding determinants that drive IPC behaviour [21]. However, it is important to note there are limited studies that are comprehensively embedded in behaviour change theories [22]. Evidence demonstrates that behaviour change interventions based on theory are more likely to be effective compared to interventions lacking theoretical underpinning [23]. Understanding how the pandemic has affected behavioural determinants of IPC practices among HCWs is important for optimising IPC improvement strategies in primary care settings. Informed by behaviour change theories, this qualitative study aimed to examine behavioural determinants shaping IPC behaviour among HCWs in general practices, reflecting on pre-, during, and post-COVID-19 pandemic.

## Methods

### Study design

For this qualitative study, semi-structured in-depth interviews were conducted during two study periods (pre-COVID-19 pandemic and during the pandemic, mainly Delta and Omicron variant periods). Since IPC implementation in general practices is strongly influenced by individual determinants for behaviour change, this study was informed by behaviour change theories that focus on individual targets. The COnsolidated criteria for REporting Qualitative research (COREQ) guidelines [24] were followed for data reporting [See Additional file 1].

### Theory

The study was informed by the Theory of Planned Behaviour [25], the Health Belief Model [26], and the Theoretical Domains Framework [27]. The Theory of Planned Behaviour suggests that behaviour is influenced by an individual’s attitudes, subjective norms, and perceived behavioural control [25]. The Health Belief Model proposes that behaviour is influenced by an individual’s perceptions of the disease severity and perceived susceptibility (together risk perception), combined with perceived benefits and barriers to the behaviour [26]. The Theoretical Domains Framework synthesises multiple behaviour change theories and is mainly developed for healthcare settings. The framework identifies twelve domains that help understand health professional behaviour, including: (1) knowledge, (2) skills, (3) professional role and identity, (4) beliefs about capabilities, (5) beliefs about consequences, (6) motivation and goals, (7) memory, attention and decision processes, (8) environmental context and resources, (9) social influences, (10) emotion regulation, (11) behavioural regulation, and (12) nature of the behaviour.

### Participants and recruitment

Participants included GPs and medical assistants from general practices mainly in the south of the Netherlands. As both GPs and medical assistants perform different IPC procedures and tasks, we aimed to recruit both professional groups to achieve broad insights into different perspectives and experiences regarding IPC.

Participants were recruited by convenience sampling with snowball methods [28, 29]. First, an invitation to participate was placed in a newsletter of the regional infection prevention and antimicrobial resistance care network to recruit potential participants. In addition, we asked participants to recruit future participants among their co-workers. We aimed to introduce diversity in our sampling in terms of participant characteristics, including different age groups, years of work experience, and sexes. In addition, we sought diversity regarding GP practice characteristics, including different sizes, types, and locations of GP practices, i.e., large and small practices, private practices and health centres (i.e., practices with multiple GPs), and practices located in both rural and urban areas. Participants were selected based on the following criteria: (a) general practitioners / medical assistants working in general practices, (b) men / women, (c) work experience more / less than 15 years, (d) working in healthcare centres / private practices, (e) working in rural / urban areas. Once HCWs expressed their willingness to participate, interviews were scheduled and an informed consent form was signed, after being provided with the study information. For those who did not respond to the initial invitations, up to three reminders were sent either via email or telephone. Recruitment of participants continued until data saturation was reached [30].

### Data collection

Data collection took place during two study periods: (1) pre-COVID-19 pandemic (hereafter named pre-pandemic): July 2019-February 2020, (2) during the COVID-19 pandemic (hereafter named during the pandemic): July 2022-February 2023 (mainly Delta and Omicron variant periods). Audio-recorded, semi-structured in-depth interviews were conducted. In-depth interviews offer the opportunity to gain detailed insights into the determinants of IPC from the perspective of the study participants, and prove particularly valuable when aiming to uncover personal experiences, beliefs, and perceptions on specific issues or topics and understand individuals’ decision-making processes [31]. These interviews were held face-to-face in the general practices where the HCWs worked. During the pandemic, a few interviews took place via video calls (due to COVID-19, there were more online meetings, so in that context, we offered the option to conduct the interviews both in person and online). The interviews pre-pandemic were held by MvH (PhD student). The interviews during the pandemic were conducted by FH (PhD student), and a second researcher was present to observe and co-guide the interviews. The presence of this second researcher provided the opportunity to collect additional data on body language and to make field notes. None of the participants were familiar with the interviewers during both data collection periods.

The interviews pre-pandemic and during the pandemic were guided by two separate topic lists, each containing open-ended questions [See Additional file 2]. Open questions were designed to encourage participants to express their feelings and thoughts freely about each question [32]. The development of these topic lists was informed by behaviour change theories including the Theory of Planned Behaviour [25], Health Belief Model [26], and the Theoretical Domains Framework [27]. The topic lists included themes as awareness/knowledge, attitudes, risk perception, professional role and identity, decision-making processes, social influence, and perceived barriers (e.g., physical environment of the GP practice and IPC materials/resources). Given our interest in examining changes in behavioural determinants as a result of the pandemic, the topic guide for the interviews during the pandemic included questions on pre-, during, and post-pandemic reflections. An example question was: “Has your thinking about IPC changed due to the COVID-19 pandemic?” In addition to these main topics, participant characteristics including age, occupation, and years of work experience were asked, as well as GP practice characteristics including the size, location, and type of GP practice.

Both topic lists were developed with input from a multidisciplinary group, including primary care professionals (e.g., GP), infection control professionals and researchers. The draft topic lists were pilot tested during the first interviews. As a result, no substantial changes were made to the interview questions, only the question order was modified.

An iterative process of data collection was employed to assess whether data saturation was reached [33], including a concurrent process of recruiting participants, collecting data, and analysing data.

### Data analysis

All interviews were transcribed verbatim by an external professional transcription service company. Transcripts were coded systematically using ATLAS.ti 9 software for qualitative analysis. Data were analysed by thematic analysis developed by Braun and Clarke [34], employing both inductive and deductive approaches. The six-step method by Braun and Clarke [34] was followed: (1) *data familiarisation*: each entire transcription was thoroughly read to gain a comprehensive understanding of the text and to familiarise the researchers with the participants’ experiences and perspectives regarding IPC in general practices, (2) *initial coding*: initial codes were derived from the data capturing the perspectives of the participants (inductive coding), while considering important concepts of behaviour change theories (deductive coding), (3) *theme generation*: extracted codes were synthesised into overarching themes, based on the topics included in the topic list and concepts from behaviour change theories (deductive coding), (4) *theme refining*: the process involved reviewing and comparing the initial codes to generate themes, (5) *theme naming*, (6) *theme interpretation*: a synthesis and overarching approach was employed to compare the qualitative findings of the two data collection periods, with careful consideration given to the connections between codes and themes of both study periods. The coding process was conducted iteratively, persisting until no additional codes emerged. In addition, field notes were compared with transcripts to develop a deeper understanding of the interpretation of the data. The field notes helped collect contextual data and identify meaningful, expressive phrases, body language, and emotions in interview passages during the coding process [35]. Thereby, our analysis focussed on both explicit and implicit dimensions of the qualitative data, providing a more nuanced and comprehensive analysis. The process of coding was independently performed by two researchers. Discrepancies during the coding process were discussed among the researchers until consensus was reached.

## Results

The study sample consisted of both GPs and medical assistants, with diversity in sex, age, and work experience. Table 1 presents the participant characteristics, for the interviews conducted pre- and during the pandemic. For the interviews pre-pandemic, 14 of the 19 invited HCWs (74%) participated. For the interviews during the pandemic, 22 of the 24 invited HCWs (92%) participated. There was overlap among participants in the initial and second study periods; 2 HCWs participated in both instances of the study. Reasons for non-participation for both data collection periods were mostly time constraints. The interviews pre-pandemic lasted on average 38 min (range 24–53 min) and the interviews during the pandemic also 38 min (range 20–53 min). For the interviews pre-pandemic, data saturation was reached after 12 interviews, and for the interviews during the pandemic, data saturation was achieved after 20 interviews.

**Table 1:** Participant characteristics of the interviews conducted pre-pandemic (*n* = 14) and during the pandemic (*n* = 22)

Participant characteristics	n (%) / M (min-max)		
	Interviews pre- pandemic (*n* = 14)	Interviews during the pandemic (*n* = 22)	
*Occupation*			
General practitioner	10 (71.4%)	13 (59.1%)	
Medical assistant	4 (28.6%)	9 (40.9%)	
*Sex*			
Female	7 (50%)	15 (68.2%)	
Male	7 (50%)	7 (31.8%)	
*Working experience (years)*	21 (1–32)	12 (6–30)	
*Age (years)*	50 (22–65)	39 (25–64)	

*Abbreviations. M =* mean, *Min =* minimum, *Max =* maximum

Qualitative analysis revealed the following themes: (1) risk perception and IPC awareness, (2) attitudes towards IPC and professional responsibility, (3) decision-making process and risk considerations for IPC adherence —these represent internal professional factors; and (4) social norm and social influence in GP practice team, (5) environmental context and resource availability in GP practice —these represent external factors. Table 2 provides an overview of the main themes and related findings, incorporating pre-, during and post-pandemic reflections. The findings are supported by illustrative quotes.

### Risk perception and IPC awareness

A major theme deriving from the interviews pre-pandemic was the generally low perceived risk of infection among GPs and medical assistants, in particular low perceived susceptibility, for both themselves and patients in general practices: *“In general, I believe that in primary care settings, there are not that many risks. Therefore, IPC measures or guidelines from secondary care settings should not be applied to our specific care setting.”* (P3, man, GP, 65-70y, pre-pandemic). Before the pandemic, participants generally expressed little desire for changes regarding IPC, as they believed they were already performing well. During the pandemic, the perceived risk of infection (both perceived susceptibility and perceived severity of disease) for patients in general practices has increased among both GPs and medical assistants, as well as the awareness of the importance of IPC: “*I am now more aware than before. I now understand what it [IPC] actually entails, the potential consequences such as severe illness and fatality, and the need for comprehensive IPC.”* (P5, woman, medical assistant, 25-30y, during the pandemic), *“Since COVID-19, our engagement with IPC has increased, it is now more integrated into our daily practice. In addition, COVID-19 has heightened awareness, particularly regarding the role patients must play in IPC. It has prompted us to consider patient compliance, and how to effectively educate them to minimise the infection risk.”* (P3, man, GP, 40-45y, during the pandemic). In contrast to the increased perceived susceptibility for patients, HCWs continued to perceive their own susceptibility to infection as relatively low, as they reported feeling protected by the precautions of wearing PPE: *“I never felt unsafe or at risk for infection myself, because I knew that I was very well protected, as the only thing I needed to do was adapt my own behaviour and wear PPE.”* (P11, woman, GP, 30-35y, during the pandemic). In addition, the majority of HCWs perceived the severity of the disease for themselves as relatively low: *“For myself, I do not see any risks. However, for the patient, I do see risks.”* (P16, woman, medical assistant, 25-30y, during the pandemic).

In the interviews during the pandemic, divergent future expectations regarding IPC awareness and risk perception emerged. On the one hand, participants expected that increased awareness would persist post-pandemic. On the other hand, participants expected that IPC awareness might decline once COVID-19 infection rates decrease due to lower perceived severity of the disease: *“As the severity of COVID-19 decreases, it will become normal again. It is a virus, just like all other flu viruses. Over time, no one will pay much attention to it [IPC] anymore.”* (P3, man, GP, 40-45y, during the pandemic).

### Attitudes and professional responsibility towards IPC

Next to positive attitudes towards IPC and its importance, the interviews pre-pandemic revealed somewhat sceptical attitudes towards IPC among a number of participants. HCWs highlighted that IPC has to be proportionate, feasible and evidence-based: *“IPC needs to remain practical and easy. We are helping people, and if things get in the way of that, it becomes irritating. So, it [IPC] should be of added value. Sometimes I wonder if it is truly progress or just nitpicking?”* (P3, man, GP, 65-70y, pre-pandemic). In addition, HCWs indicated to sometimes question the importance of certain IPC measures: *“Some rules make you think, does it all need to be so strict? We just received a new IPC guideline, which states that we should use disposable gowns for minor procedures. I question whether there is room for improvement in that area and why we should implement even more measures*.” (P6, woman, GP, 45-50y, pre-pandemic). This attitude was associated with beliefs about consequences (i.e., outcome expectation), specifically related to what is known as the ‘prevention paradox’: *“Typically, immediate consequences of non-compliance with IPC are not observable, which may lead to a lack of motivation to change your behaviour.”* (P10, man, GP, 60-65y, pre-pandemic). In addition, both pre- and during the pandemic, a few HCWs perceived IPC to take a lot of time: “*IPC takes a lot of time, and I must confess, I am not particularly fond of tasks that require a lot of time.”* (P5, woman, GP, 45-50y, pre-pandemic). In the interviews during the pandemic, some HCWs doubted the effectiveness of certain IPC measures: “*Wearing a disposable gown is truly ineffective, as you would need to have snot on it [gown] and lick it off to spread the infection.”* (P12, woman, GP, 45-50y, during the pandemic), *“I heard from a reliable source that disinfectant is not effective in killing the coronavirus.”* (P4, woman, GP, 60-65y, during the pandemic). Moreover, HCWs sometimes indicated the inconvenience of using PPE, in both the interviews pre- and during the pandemic: “*A blue disposable gown is very hot, it is very inconvenient and not comfortable. Within just five minutes of wearing it, I become quite sweaty. User-friendliness is definitely an issue.”* (P3, man, GP, 40-45y, during the pandemic). The inconvenience of wearing PPE was often associated with the desire to deliver good patient care: *“Wearing a mask is unpleasant, especially during patient consultations. It limits the visibility of facial expressions, making it difficult for patients with hearing impairments to understand me. While patients keep their masks on, I sometimes need to remove mine for better communication.*” (P1, woman, GP, 50-55y, during the pandemic).

Both pre- and during the pandemic, the majority of HCWs indicated that compliance with IPC is part of their professional responsibility to deliver good patient care, i.e., protect patients from avoidable infections. Participants indicated that next to complying with IPC, their professional responsibility also includes providing patient education on IPC. Nonetheless, several HCWs indicated that IPC measures should be in balance with other aspects of care delivery: *“Next to IPC, we have patient care and acute patient care to provide. Our profession is broader than just IPC. If too much focus is placed solely on IPC, it comes at the expense of other parts of care provision. Therefore, it is crucial to consider the feasibility of IPC measures in the work context while ensuring that all other aspects of care are prioritised.”* (P1, woman, GP, 50-55y, during the pandemic). Moreover, GPs expressed the need for autonomy in making IPC-related decisions, as making autonomous decisions is part of their professional identity. The decision-making process and risk considerations of GPs are important herein.

### Decision-making process and risk considerations for IPC adherence

During the pandemic, the decision of whether to adhere to IPC measures was dependent on the individual GP and influenced by multiple factors and the GP’s risk assessment. The main reasons stated by GPs and medical assistants for adhering to IPC were to protect the patient (with extra vigilance for vulnerable groups such as the elderly population and immunocompromised patients), protect themselves to prevent staff absenteeism (and ensure continuity of care), and protect their household and relatives. The decision to adhere to IPC was primarily influenced by the nature of the consultation (duration, level of physical contact, and setting), the type/risk indication of the patient (respiratory symptoms/COVID-19 suspicion and vulnerability of the patient group), and the ability to provide good patient care (including effective communication). The season (e.g., flu season), community prevalence or incidence, and the pathogenicity of the virus also contributed to risk considerations and the decision-making process for adhering to IPC measures.

*“Factors depend on the patient’s vulnerability and perceived likelihood of COVID-19 infection. Setting matters, whether it is a consultation at the practice or home visit, and the ability to maintain distance, and the duration of the consultation. Next to the patient, the risk of exposure to infections is crucial, whether there is a norovirus or tuberculosis outbreak or high community incidences of COVID-19, which also depends on the season.”* (P7, man, GP, 30-35y, during the pandemic), *“At times, catering to patient needs is essential. If they cannot understand me without seeing my mouth, I remove my face mask. Clear communication is vital between physician and patient.”* (P2, woman, GP, 35-40y, during the pandemic).

Additionally, sustainability emerged as an important consideration. Participants often indicated that IPC (particularly disposable PPE) conflicts with sustainability goals: *“Environmental and sustainability considerations are important. We should integrate environmental considerations into IPC. For example, we started using washable white coats instead of disposable plastic gowns, which we wash at 60 degrees Celsius. This should be adopted more widely, particularly to ensure a future for the next generation.”* (P12, woman, GP, 45-50y, during the pandemic).

In addition to general decision-making processes and risk considerations, HCWs were asked for their specific considerations and the decision-making process related to the upscaling of IPC measures in general practices. Most HCWs indicated that scaling up is determined by a combination of internal (within the GP practice) and external (outside the GP practice) factors. External factors encompass guidelines and recommendations from the government, the professional association (NHG), and public health services. Additionally, external factors involve increasing infection rates and community incidence, disease burden (determined by the pathogenicity of the virus), and rising hospital occupancy. Internal factors that were mentioned as reasons for scaling up IPC measures in the GP practice included increasing infections among the patient population and among staff to prevent further personnel shortages.

*“It is important to assess the severity of illness, disease burden, age groups affected, and hospitalisation rates. When hospital admissions rise, scaling up measures is necessary, particularly in the face of potential staff shortages. The decision to scale up IPC measures is influenced by various factors, including external factors such as hospital bed availability and governmental policies, as well as internal factors like potential staff shortages.”* (P5, woman, medical assistant, 25-30y, during the pandemic).

### Social norm and social influence in GP practice team

Both pre- and during the pandemic, the social context emerged as an important factor influencing IPC behaviour. In the interviews pre-pandemic, HCWs indicated that IPC was often part of the social norm in the team, in particular the injunctive norm: *“We all have the same views on IPC, collectively considering its importance. Everyone is on the same page.”* (P7, woman, medical assistant, 20-25y, pre-pandemic). In addition, exemplary behaviour, the process of social norms, was indicated to influence IPC behaviour: *“I wash my hands frequently and even disinfect them afterwards. Now my colleagues do that as well. Previously, it was less common. They started doing it because they see me doing it.”* (P1, woman, GP, 30-35y, pre-pandemic). The influence of motivated HCWs (i.e., internal change coaches) was also mentioned to enhance IPC motivations, and therefore behaviour: *“My colleagues often tell me that my enthusiasm rubs off on them, and that they try to be as enthusiastic as I am.”* (P10, man, GP, 60-65y, pre-pandemic). In the interviews during the pandemic, participants frequently mentioned that IPC agreements and work practices were established through shared decision-making in the team and mutual agreement: *“We always discussed IPC measures in team meetings, with other GPs and medical assistants.”* (P4, woman, GP, 60-65y, during the pandemic). This created a sense of shared responsibility and ownership among HCWs in the team.

### Environmental context and IPC resource availability in GP practice

During the pandemic, HCWs primarily reported barriers related to the GP practice building (i.e., physical structure) and layout (i.e., spatial arrangement of the practice), as well as limited IPC resource and material availability: “*During the first waves of the pandemic, resource and equipment scarcity was a concern, especially regarding PPE and tests. Additionally, facility building and layout barriers are important, including barriers like small waiting areas or inadequate facility layout that hinders the establishment of separate entrances and exits.”* (P7, man, GP, 30-35y, during the pandemic). Multiple participants reported that the building was inadequate for managing patient flow, maintaining (1.5-meter) physical distancing in the waiting room, and ensuring proper ventilation: “*We had a lack of space in the waiting room, leading to barriers to accommodate people while adhering to the 1.5-meter physical distancing requirement. Consequently, we had to ask patients to wait outside, a situation that was far from ideal, especially in colder weather.”* (P2, woman, GP, 35-40y, during the pandemic), *“Our ventilation options are limited. Despite having numerous windows in our building, regrettably, they are all sealed shut and cannot be opened.”* (P6, woman, medical assistant, 35-40y, during the pandemic). Moreover, GP practice characteristics such as the type and size of the practice played a role. HCWs working in health centres and larger practices reported experiencing additional hinderances: “*We have a large team of 25 people, including many physicians, assistants, and support staff. Maintaining consistent communication and adherence to national guidelines is a challenge due to the size of the team. It is difficult to reach everyone simultaneously and ensure uniform triage and harmonise IPC practices.”* (P11, woman, GP, 30-35y, during the pandemic), “*An important barrier was that I have a practice within a health centre. If I were to make certain decisions, such as closing the front door, it would also prevent other patients from accessing physiotherapy, skincare treatments, and other healthcare services provided in this building.”* (P15, man, GP, 45-50y, during the pandemic).

In the interviews pre-pandemic, HCWs also expressed concerns about the limited availability and access to IPC materials and equipment, such as disinfection dispensers. Participants also noted a lack of organisational commitment to IPC, highlighting that IPC generally only received attention in light of accreditation requirements or quality assurance.

**Table 2:** Overview of main themes and related findings, incorporating pre-, during, and post-pandemic reflections, reported by general practitioners and medical assistants (*n* = 14, interviews pre-pandemic; and *n* = 22, interviews during the pandemic)

Theme	Findings and reflections pre-, during, and post-pandemic
*Internal professional factors*	
Risk perception and IPC awareness	*Pre-pandemic* Relatively low perceived risk of infection^a^ for both patients and HCWs in general practices *During the pandemic* Increased perceived risk of infection for patients and awareness of the importance of IPC Relatively low perceived risk of infection for HCWs themselves *Future expectation (post-pandemic)* Diverse future expectations: one group expected IPC awareness to remain heightened post-pandemic, while others expected IPC awareness to decline once COVID-19 infection rates decrease (lower perceived severity of the disease)
Attitudes towards IPC and professional responsibility^b^	*Pre-pandemic* IPC part of professional responsibility (protect patients from avoidable infections) IPC has to be practical, of added value, and evidence-based IPC takes a lot of time (perceived time investment) Balancing IPC with other aspects of patient care and professional roles Autonomy in making IPC-related decisions *During the pandemic* IPC part of professional responsibility (protect patients from avoidable infections) Doubts about the effectiveness of certain IPC measures IPC takes a lot of time (perceived time investment) Balancing IPC with other aspects of patient care and professional roles Autonomy in making IPC-related decisions
Decision-making process and risk considerations for IPC adherence	*During the pandemic* Main reasons for HCWs to adhere to IPC: (1) protect the patient (with extra vigilance for vulnerable groups such as the elderly population and immunocompromised patients), (2) protect themselves to prevent staff absenteeism (and ensure continuity of care), and (3) protect their household and relatives The decision to adhere to IPC was influenced by multiple factors and risk assessments: • the nature of the consultation (duration, level of physical contact, and setting, i.e., home visit or consultation at GP practice); • the type/risk indication of the patient (respiratory symptoms/COVID-19 suspicion and vulnerability of the patient group); • the season (e.g., flu season), community prevalence or incidence; • the pathogenicity of the virus; • ability to provide good patient care (including effective communication); • sustainability considerations Decision-making and considerations to upscale IPC measures were influenced by: • Internal factors (within GP practice): increasing infections among the patient population and among staff (to prevent further personnel shortages); • External factors (outside GP practice): guidelines and recommendations from the government, the professional association (NHG), and public health services; increasing infection rates and community incidence; increasing disease burden (based on pathogenicity of the virus), and rising hospital occupancy
*External factors*	
Social norm and social influence in GP practice team	*Pre-pandemic* Social norms (injunctive norm, descriptive norm), exemplary behaviour, and internal change coaches reinforce IPC behaviour *During the pandemic* IPC discussed in team meetings, shared decision-making, and mutual agreement in team
Environmental context and IPC resource availability in GP practice	*Pre-pandemic* • Resources and materials: limited availability and access to IPC materials and equipment • Lack of organisational commitment to IPC (IPC generally only received attention in light of accreditation requirements or quality assurance) *During the pandemic* • GP practice building and layout: inadequate practice building or layout affecting patient flow, physical distancing and adequate ventilation • Resources and materials: limited IPC resource and material availability (PPE and tests), particularly during the first waves of the pandemic • Other barriers include the large size of the GP practice (e.g., health centres)

*Abbreviations. IPC =* infection prevention and control, *GP* = general practitioner, *HCWs =* healthcare workers, *PPE =* personal protective equipment

^a^Perceived risk of infection includes both perceived susceptibility and perceived severity of disease

^b^The interviews also revealed positive attitudes towards infection prevention and control (IPC). However, for this study, our primary focus was on exploring modifiable factors

## Discussion

Informed by behaviour change theories, this qualitative study examined behavioural determinants shaping IPC behaviour among HCWs in general practices, reflecting on pre-, during, and post-COVID-19 pandemic. By assessing behavioural determinants, we gain insights into the factors influencing HCWs’ adoption of IPC practices. Understanding these factors enables the development of strategies aimed at promoting HCWs’ adoption and adherence to IPC practices. Our results demonstrated increased risk perception and awareness towards the importance of IPC during the pandemic compared to the pre-pandemic period, with diverse future expectations (post-pandemic) regarding IPC awareness. A consistent belief emerged both pre- and during the pandemic that IPC is part of professional responsibility, while needing to be balanced with other aspects of patient care. Findings revealed that decision-making is dependent on the individual GP and is mainly influenced by risk assessments and sustainability considerations. In addition, the social context in the practice team can reinforce IPC behaviours. GP practice building and layout, and limited IPC resource and material availability were reported as main barriers.

The risk perception and awareness of the importance of IPC among HCWs reported in this study parallels findings of previous international studies, which have demonstrated advances in implementing IPC measures among HCWs during the pandemic compared to the pre-pandemic period [8, 14–16]. A previous review including studies across different healthcare settings has suggested that the heightened risk of infection and outbreaks motivates HCWs to enhance their IPC practices, driven by fear and increased recognition of the significance of adhering to IPC protocols [17]. Previous qualitative findings in Belgian general practices have indicated that during the pandemic, GPs perceived themselves as working in a high-risk setting [11]. Notably, their primary concern was not their own susceptibility to illness, but rather their inability to continue care provision due to becoming ill themselves, which aligns with our findings. The findings of our study that decision-making is dependent on the individual GP is in accordance with previous qualitative studies, which have indicated that IPC implementation is highly influenced by the preferences and needs of individual GPs, leading to autonomous decision-making [8, 36]. In addition, a previous review including studies in various healthcare settings has indicated the important role of professional responsibility and the desire to deliver good patient care as factors that influence HCWs’ IPC behaviour [37], which is in line with the findings of the present study. Another review study has shown that HCWs occasionally perceived the use of PPE to be inconvenient, and to have a negative impact on patient care, particularly in terms of physician-patient communication [17]. Furthermore, both in the Netherlands and internationally, there is increasing attention to sustainability in healthcare, including general practices [38–40]. This may explain GPs becoming more aware of the need to consider sustainability in their decision-making processes. In line with our findings, previous review studies including studies in different healthcare settings have stressed the enabling function of the social context—such as the social norm—in the team on IPC behaviour [17, 22]. This indicates that individuals are more likely to adhere to these practices, creating a culture of IPC and responsibility, when IPC practices are socially approved and perceived as the norm within a team. Nevertheless, a potential hindering role of the social context was also suggested in previous studies, in terms of observed non-compliance and negative modelling [17]. This can undermine IPC behaviours, resulting in a culture of non-compliance and diminished effectiveness in IPC efforts. Moreover, a previous study has indicated the positive influence of shared decision-making regarding IPC and practice policy in professional teams during the pandemic, and highlighted the importance of continuing this in primary care post-pandemic [41]. Our findings regarding the reported barriers by HCWs concerning the GP practice building and layout, and limited IPC resource and material availability corroborate previous studies that have identified major problems with the availability of PPE including medical masks during the pandemic [17, 42]. In addition, a previous study conducted before the pandemic has indicated the barrier of limited space and time constraints for adequate IPC practices in primary care settings [43]. The same study has reported that inspection by health authorities and fear of legal action were drivers for positive behavioural change. This is in line with our findings from the interviews pre-pandemic, which indicated that IPC mainly received (organisational) attention in light of accreditation requirements. In addition to individual professional, social, and environmental factors influencing HCWs’ IPC behaviour, it is important to recognise that IPC may also be influenced by contextual factors relating to the pandemic and the healthcare sector such as legislative and regulatory frameworks, health system infrastructure and logistical factors [8, 44].

### Strengths and limitations

The strength of this study lies in its underpinning by multiple behaviour change theories, thereby providing comprehensive insights into factors influencing IPC behaviour. The effectiveness of theory-based interventions increases as the number of relevant incorporated theories is greater [45]. Next to deductive approaches, we also employed inductive approaches for data analysis, to integrate theories while also remaining open to new insights [46]. Moreover, we were able to compare perspectives of IPC among HCWs in general practices both before and during the pandemic, which provides additional understanding of this topic. This study is to the best of our knowledge the first study of this nature conducted in a setting of general practices.

The present study also has several limitations. First, this study employed convenience sampling techniques to recruit participants, which has the potential to recruit participants with a higher willingness and therefore introduce selection bias [47]. However, this study included a diverse sample of HCWs with diversity in occupation, sex, age, and years of working experience, as well as diversity in GP practice characteristics, including size, type, and location of GP practices. Hereby, minimising the anticipated impact of selection bias. The recruitment of GPs and medical assistants for interviews was challenging, primarily due to demanding workloads, which were further intensified during the pandemic. Of note, this challenge and other contextual factors such as staff turnovers and staff shortages resulted in the inability to approach the same individuals in both study periods. Second, recall bias may be a potential concern in retrospective reflections on the pandemic period, as most interviews were conducted during periods of lower burden of disease compared to the initial waves of the pandemic. Nevertheless, we anticipate this bias to be minimal due to the relatively recent and impactful nature of the pandemic, presumably resulting in a more vivid memory recall. Additionally, the impact of newly implemented IPC measures, such as the use of protective screens, face masks and disposable gowns, which were not extensively used before, probably further contributed to enhanced recall of HCWs. One should note that our study was conducted mainly in the south of the Netherlands. While this geographical focus may limit generalisability, we anticipate that our findings offer insights applicable across the Netherlands and potentially other Western countries.

### Implications for practice

With the findings of this study, we aim to underscore the important lessons learned from the COVID-19 pandemic, highlighting the importance of maintaining IPC awareness and preparedness for infectious disease outbreaks post-pandemic. Research indicates that employing multifaceted or multimodal approaches enhances the effectiveness of interventions aimed at optimising IPC in healthcare settings [48, 49].

Our findings indicated a concern that after the pandemic, the sense of urgency for IPC and IPC awareness may decrease. As the importance of IPC extends beyond pandemic contexts and remains critical during seasonal respiratory epidemics (flu season), maintaining IPC awareness and positive attitudes towards IPC is important. In particular, fostering a positive attitude towards IPC by emphasising its integral part of professional responsibility in ensuring a safe care environment and protecting patients from preventable infections is essential. Additionally, altering beliefs that IPC requires additional time is also important. To achieve this, ongoing education targeting the identified modifiable determinants is recommended [50]. Methods may include interprofessional education during team meetings, and regular updates on IPC guidelines and best practices through newsletters or emails [41]. Persuasive communication appeals may effectively convey the risks associated with inadequate IPC compliance [51]. In addition, sharing real-life examples and experiences can be beneficial, as personal stories can make the importance of IPC more tangible [52]. A previous study examining the effectiveness of communication strategies regarding IPC in acute care settings has highlighted the need to tailor communication to different groups of HCWs – especially for (digital) communications aimed at larger audiences – and to involve HCWs in developing communication strategies [53].

Acknowledging the important role of IPC beyond the pandemic context, HCWs should integrate IPC practices into their daily work routines, eliminating the belief that IPC requires additional time and effort and aligning it with other aspects of primary care delivery. This integration demands both individual behavioural change and organisational effort. Organisations should embed interventions to optimise IPC within their ongoing workflow and quality assurance initiatives to ensure sustained IPC practices. The use of reminders, behavioural nudges (e.g., strategic placement of hand sanitiser dispensers), and frequent IPC education and training—including peer (interprofessional) education—could be approaches herein [50]. Previous studies have shown the effectiveness of electronic reminder systems in improving hand hygiene practices [54], and hands-on learning that integrates HCWs’ professional experiences in enhancing IPC behaviour [48, 55].

In our study, HCWs reported the need for a balance between IPC and other aspects of primary care delivery, which indicates that IPC improvement strategies should be tailored and compatible with professional roles and different aspects of care delivery. For this, it is important to consider the unique (risk) considerations that HCWs — particularly GPs — weigh during patient care. A recommendation would be to refine existing IPC guidelines and protocols by incorporating decision-making tools (e.g., decision tree or checklist). These support tools can assist HCWs in making comprehensive and well-rounded decisions that encompass thorough risk considerations or assessments. In addition, these decision-making tools can include sustainability considerations. The goal is to support HCWs in risk assessments and selecting appropriate measures in certain situations. By doing so, HCWs can make informed choices that consider patient safety and other relevant factors of patient care provision. This becomes particularly relevant as our findings indicated that the decision-making process regarding IPC behaviour is dependent on individual GPs, making such tools valuable in harmonising risk considerations among GPs and other HCWs in general practices. A previous study has also indicated the importance of the development and implementation of clinical decision-making tools for pandemic preparedness of primary care [56].

Our findings demonstrated the influential role of the social context, social approval, and normative behaviour in driving IPC behaviour. Therefore, fostering an IPC culture at the team and organisational level should be strived for. Modelling is suggested to be an effective behaviour change method to enhance IPC behaviour [22]. Therefore, it is recommended to make (experienced) GPs aware of their role modelling function and motivate them to practice adequate IPC behaviour. In addition, specific methods such as regular (interdisciplinary) team meetings, where insights, knowledge, experiences, and best practices are shared can foster a culture of collaboration and knowledge-sharing, and contribute to enhanced IPC behaviours [41, 57]. A lack of these meetings has been noted to diminish job satisfaction, and increase workload and burnout, thereby indirectly posing a risk to patient safety [58]. The collaborative nature of decision-making during the pandemic in regular team meetings, and associated ownership and shared responsibility, indicates the value of continued teamwork and interprofessional communication post-pandemic. A previous review has highlighted that interventions to enhance IPC behaviour rarely include group- or team-oriented strategies, and mainly focus on strategies aimed at individuals and organisational aspects [22].

Our findings identified the physical environment of the GP practice and resource availability as barriers to IPC behaviour. This indicates the need for organisations to proactively ensure the availability of necessary resources and IPC equipment. Nevertheless, since the availability of resources is influenced by higher-level systemic factors (e.g., supply chains or governmental regulations), it is important for regional authorities and healthcare organisations to facilitate and coordinate resource allocation on a broader scale, for example on the regional level. In addition, the GP practice building and layout should enable IPC measures such as regulating patient flow, physical distancing, and ensuring adequate ventilation systems and sufficient (natural) ventilation opportunities. Therefore, it is recommended for future building planning to consider these aspects when building new GP practice facilities. Furthermore, current GP practices should consider (if possible) facility redesign to optimise IPC and enhance preparedness for future epidemics or pandemics. This can involve rearranging facility layout to improve patient flow patterns, assigning specific areas (including waiting areas) for infectious patients, and improving ventilation systems.

## Conclusions

The findings of this study offer actionable recommendations for promoting sustained IPC behaviours in general practices. A multifaceted approach that focuses on continuous education using targeted communication strategies, IPC integration into HCWs’ work routines and organisational workflows, fostering a culture of IPC in the practice team through knowledge-sharing and teamwork, and addressing GP practice physical environment and IPC resource barriers is recommended for optimising IPC behaviour. Moreover, to enhance informed decision-making, harmonise professional decision-making, and balance IPC requirements and other professional roles and responsibilities, refining existing IPC protocols through the incorporation of decision-making tools is advised.

### Electronic supplementary material

Below is the link to the electronic supplementary material.

Supplementary Material 1

Supplementary Material 2

## Data Availability

Data can be made available on request to the head of the data-archiving of the Public Health Service South Limburg. Interested researchers should contact the head of the data-archiving of the Public Health Service South Limburg (Tamara Kleine: tamara.kleine@ggdzl.nl) when they would like to re-use data.
