# Supplementary material for: Behavioural determinants shaping infection prevention and control behaviour among healthcare workers in Dutch general practices: a qualitative study reflecting on pre-, during and post-COVID-19 pandemic
Source: BMC Prim Care. 2024 Feb 28;25:72. doi: 10.1186/s12875-024-02304-9 (PMC10900587; doi:10.1186/s12875-024-02304-9)
Supplement: Supplementary file 2 — Supplementary Material 2 [file 12875_2024_2304_MOESM2_ESM.pdf]

Additional file 2: Interview topic guides for the interviews pre- and during the COVID-19 pandemic

Topic guide for the interviews pre-pandemic

**Background variables**	1. Could you introduce yourself?<br>o Age, gender, occupation, years of (work) experience.
	2. Could you tell me about your general practice (or the practice where you work)?<br>o Do you work in a solo practice or a group practice?<br>o Are there specific patient groups you have more contact with than others?
**Behavioural determinants**	3. What comes to mind when you think about infection prevention and control (IPC)?<br>o To what extent do you consider IPC important? [attitude]<br>o Could you share some experiences with IPC (measures)?
	4. To what extent does IPC play a role at work?<br>o What do you think your colleagues think of (the importance of) IPC? [social norm]<br>o To what extent do colleagues monitor/correct each other on IPC practices? [social influence]<br>o To what extent do you encourage patients to apply IPC measures?
	5. To what extent does IPC play a role at an organisational level? (e.g., policy, formal agreements).<br>o How much attention is given to IPC?<br>o Do you think enough priority is given to IPC?<br>o Who deals with/are involved in IPC in your organisation?<br>6. Do you experience barriers/challenges in performing IPC? If yes, which ones?
**Additional comments**	7. Do you have any (additional) questions or comments for us? Or additional experiences or opinions to share?

Abbreviation: IPC infection prevention and control

<u>Topic guide for the interviews during the pandemic</u>

**Introduction**	1. Could you introduce yourself?<br>o Age, gender, occupation, educational background, years of (work) experience.
	2. Could you tell me about your general practice (or the practice where you work)?<br>o Do you work in a solo practice or a group practice?<br>o Are there specific patient groups you have more contact with than others?
**Behavioural determinants**	3. What comes to mind when you think about infection prevention and control (IPC)?<br>o What do you understand by IPC, what does it entail? [awareness/knowledge]<br>o What are your thoughts on IPC? To what extent do you consider IPC important? [attitude]<br>o What role has IPC in your professional role? [professional role]<br>o Has your thinking about IPC changed due to the COVID-19 pandemic? [attitude/awareness/risk perception]<br>▪ How do you think this will be post-pandemic?<br>4. What do you think your colleagues think of (the importance of) IPC? [social norm]<br>5. What are the reasons for you to perform IPC? [decision-making]<br>o What considerations are made in this regard? [risk considerations]<br>o What factors play a role in the decision to perform or not to perform certain IPC measures?<br>6. Do you experience barriers/challenges in performing IPC? If yes, which ones?

	o Did the barriers/challenges you experience change due to the COVID-19 pandemic?
**Additional comments**	7. Are there any other things/experiences you would like to share regarding IPC?<br>o Are there any other points we have not discussed that you would like to address?<br>o Do you have any further questions or comments for us?
Abbreviation: IPC infection prevention and control	
